# Circulating Total Extracellular Vesicles Cargo Are Associated with Age-Related Oxidative Stress and Susceptibility to Cardiovascular Diseases: Exploratory Results from Microarray Data

**DOI:** 10.3390/biomedicines11112920

**Published:** 2023-10-28

**Authors:** Laura Reck Cechinel, Rachael Ann Batabyal, Giana Blume Corssac, Madeleine Goldberg, Brennan Harmon, Virgínia Mendes Russo Vallejos, Gisele E. Bruch, André Ricardo Massensini, Adriane Belló-Klein, Alex Sander da Rosa Araujo, Robert J. Freishtat, Ionara Rodrigues Siqueira

**Affiliations:** 1 Programa de Pós-Graduação em Ciências Biológicas: Fisiologia, Universidade Federal do Rio Grande do Sul, Porto Alegre 90035-003, RS, Brazil; lreck@childrensnational.org (L.R.C.);; 2 Center for Genetic Medicine Research, Children’s National Research Institute, Washington, DC 20012, USA; 3 Division of Emergency Medicine, Children’s National Hospital, Washington, DC 20010, USA; 4 School of Medicine and Health Sciences, The George Washington University School of Medicine and Health Sciences, Washington, DC 20052, USA; 5 Laboratório de Fisiologia Cardiovascular e Espécies Reativas do Oxigênio, Departamento de Fisiologia, Instituto de Ciências Básicas da Saúde, Universidade Federal do Rio Grande do Sul, Porto Alegre 90035-003, RS, Brazil; 6 Departamento de Fisiologia e Biofísica, Instituto de Ciências Biológicas, Universidade Federal de Minas Gerais, Belo Horizonte 31270-901, MG, Brazil; 7 Departamento de Farmacologia, Instituto de Ciências Básicas da Saúde, Universidade Federal do Rio Grande do Sul, Porto Alegre 90035-003, RS, Brazil

**Keywords:** aging, extracellular vesicles and particles, cardiac aging, NADPH oxidase, redox homeostasis

## Abstract

Aging is a risk factor for many non-communicable diseases such as cardiovascular and neurodegenerative diseases. Extracellular vesicles and particles (EVP) carry microRNAs that may play a role in age-related diseases and may induce oxidative stress. We hypothesized that aging could impact EVP miRNA and impair redox homeostasis, contributing to chronic age-related diseases. Our aims were to investigate the microRNA profiles of circulating total EVPs from aged and young adult animals and to evaluate the pro- and antioxidant machinery in circulating total EVPs. Plasma from 3- and 21-month-old male Wistar rats were collected, and total EVPs were isolated. MicroRNA isolation and microarray expression analysis were performed on EVPs to determine the predicted regulation of targeted mRNAs. Thirty-one mature microRNAs in circulating EVPs were impacted by age and were predicted to target molecules in canonical pathways directly related to cardiovascular diseases and oxidative status. Circulating total EVPs from aged rats had significantly higher NADPH oxidase levels and myeloperoxidase activity, whereas catalase activity was significantly reduced in EVPs from aged animals. Our data shows that circulating total EVP cargo—specifically microRNAs and oxidative enzymes—are involved in redox imbalance in the aging process and can potentially drive cardiovascular aging and, consequently, cardiac disease.

## 1. Introduction

Aging is a risk factor for several non-communicable diseases [1]. With the aged population continuing to grow, concerns regarding public health and socioeconomics have also escalated. To address these concerns, it is necessary to gain a clear understanding of the complex biological processes that underlie the biochemical and physiological changes associated with aging; this includes the spread of molecules involved in both normal and impaired aging processes, which may create a permissive environment that increases susceptibility to age-associated diseases.

In this context, extracellular vesicles and particles (EVPs) may play a role in spreading molecules involved with oxidative stress in the aging process. Circulating EVPs obtained from aged Wistar rats (21- and 26-month-old) have higher reactive species levels [2] in addition to a reduced content of tetraspanin CD63, a marker of extracellular vesicles [2,3]. Interestingly, this finding provides further support for Harman’s [4] free radical theory of aging, which proposes that oxidative stress plays a crucial role in the natural aging process and the development of age-related diseases [4], and since then, it has been widely accepted [5,6,7,8].

Despite these findings, it is not known whether the increased reactive species found in circulating total EVPs from aged animals are generated naturally within the EVPs. As mentioned, EVPs are a potential mechanism by which molecules involved in oxidative stress are spread [9]. Borras and colleagues [10] proposed an age-related imbalance of EVP pro- or antioxidant cargo in a recent review; however, they did not directly examine oxidative stress parameters in EVPs during the normal aging process. The presence of NADPH oxidase subunits such as NOX2 in EVPs [11,12,13] and their association with increased superoxide anion levels in blood-derived EVPs from septic patients [11] suggests a possible link between NADPH oxidase (NOX2) and age-induced reactive species content in circulating EVPs. NOX2 and myeloperoxidase (MPO) are two enzymes that play important roles in cardiovascular diseases [14,15]. Another enzyme source of reactive species, xanthine oxidase (XO), as well as the antioxidant system—namely, catalase (CAT) —have not been studied in EVPs during aging but may play important roles in the age-related disruption of redox homeostasis. 

In addition to enzymes, tetraspanins, and other molecules, EVPs carry microRNAs (miRNNAs). The role of miRNAs transported by circulating EVPs has received attention in the aging process. Lee and colleagues (2018) reported that middle-aged mice (12-month-old) show changes in exosomal miR-126b-5p, miR-184-3p, and miR-200b-5p levels [16]; however, the impact of aging on circulating EVP miRNA cargo from rats has not been properly evaluated. An intricate relationship may exist between oxidative stress and total EVP miRNA signatures in both the aging process *per se* and in age-related diseases. For example, in vitro models show that oxidative stress can alter miRNA expression [17]. Engedal and colleagues [18] reviewed published findings on oxidative stress-dependent miRNA expression and, using in silico tools, identified several oxidative stress-modulated miRNAs, their predicted mRNA gene targets, and the affected canonical pathways. 

This study aimed to investigate the effects of the aging process on miRNA signatures in circulating total EVPs; and to perform an in silico analysis of their downstream signaling pathways to predict impacts on physiological and biochemical functions. In addition, we aimed to evaluate redox homeostasis by determining the levels of reactive species-generating enzymes and an antioxidant defense enzyme in circulating total EVPs. 

## 2. Materials and Methods

### 2.1. Animals

The experiment used male Wistar rats at two different ages, 3 months and 21 months, provided by the Centro de Reprodução e Experimentação de Animais de Laboratório (CREAL) and kept under standard conditions. The experiment followed the NIH guidelines for animal care and use, and it was approved by the Local Ethics Committee (CEUA—Comissão de Ética no Uso de Animais—UFRGS; nr.29818). The animals were decapitated in the morning, and trunk blood was collected. Plasma samples were obtained by centrifugation (1200× *g* for 10 min). All samples (young adult and aged) were processed equally to minimize technical errors and bias, as follows: plasma was frozen immediately after collection at −80 °C, and once thawed, the EVPs were immediately isolated and subsequent analysis was performed [19].

### 2.2. Circulating Extracellular Vesicle and Particle Isolation and Characterization

EVP isolation was performed using a commercial precipitation solution (ExoQuick^TM^ System Bioscience, Mountain View, CA, USA), following the manufacturer’s instructions. The total protein content of circulating EVPs was measured by the Coomassie blue method, using bovine serum albumin as the standard [20]. The circulating EVPs were characterized by their size distribution using Nanoparticle Tracking Analysis (NTA—NanoSightLM14 and NTA 3.2 Analytical Software; Nanosight Ltd., Amesbury, UK) and by Western blotting of flotillin1 (FLOT1). 

### 2.3. microRNA Analysis

microRNA was isolated from total EVPs using the mirVana microRNA isolation kit (Life Technologies, Carlsbad, CA, USA) and amplified with the SeraMir Exosome RNA Amplification Kit (System Biosciences, Mountain View, CA, USA), according to the manufacturer’s instructions. RNA was labeled with an Affymetrix^®^ FlashTag™ Biotin HSR RNA Labeling Kit (Affymetrix, Santa Clara, CA, USA), according to standard procedures. Labeled microRNA was hybridized to Affymetrix GeneChip microRNA 4.0 arrays. The resulting data were analyzed in Expression Console using RMA+DMBG normalization (Affymetrix, Santa Clara, CA, USA), then exported to Partek Genomics Suite (Partek, St. Louis, MO, USA) for analyses.

### 2.4. Western Blot for Pro- and Antioxidant Machinery

Western blotting was performed to evaluate the levels of gp91phox (NADPH oxidase subunit, NOX2), XO, CAT, and FLOT1 (*n* = 6 animals per group) in EVPs. Samples were prepared (40–80 μg/protein) using RIPA and Laemmli buffers, separated by SDS-PAGE (SDS-PAGE, 1.5 mm, 130 volts), and transferred to PVDF membranes (Millipore, Burlington, MA, USA). The membranes were then incubated with the primary antibodies anti-gp91phox (abcam, Cambridge, MA, USA), anti-CAT, anti-XO (Santa Cruz Biotechnology, Santa Cruz, CA, USA) and anti-FLOT1 (System Biosciences, Palo Alto, CA, USA), and subsequently with a secondary antibody (Santa Cruz Biotechnology, Santa Cruz, CA, USA); the molecular weights of the bands were determined using a molecular weight marker (RPN 800 rainbow full range Bio-Rad, CA, USA). The Ponceau method was used for normalization [21,22,23].

### 2.5. Determination of Pro- and Antioxidant Enzyme Activities

To analyze enzyme activity, EVPs were lysed with Triton X-100 at a final concentration of 0.1% [24]. For the MPO enzymatic reaction, a mixture of potassium phosphate buffer (0.1 M), EVPs, and 2% ortho-dianisidine was prepared. H_2_O_2_ (0.01%) was then added, and MPO activity was determined by absorbance. XO activity was measured according to the method of [25], where activity is measured by determining uric acid levels from xanthine. CAT activity was measured using the method described by Aebi [26]. The MPO and CAT activity results were expressed in percentage of control. The control group (young adult) was considered to represent 100% enzymatic activity. 

### 2.6. Statistical Analysis

To address the changes between the aged and young adult groups, we performed a Student’s *t*-test with GraphPad Prism v.6 (Boston, MA, USA). Differences with *p*-values less than 0.05 were considered significant. To identify differentially expressed miRNAs between the groups, we performed a one-way analysis of variance with Partek Genomics Suite (version 6.6, Partek, St. Louis, MO, USA). miRNAs that were statistically significant between the groups (*p* < 0.05) and had a fold change ≥ |1.1| were selected for further analysis.

Differentially expressed miRNAs were analyzed with QIAGEN Ingenuity Pathway Analysis (version 94302991, QIAGEN Inc., Redwood City, CA, USA https://digitalinsights.qiagen.com/IPA - accessed on 25 September 2023) [27] to investigate the effects of miRNAs on gene expression regulation during the aging process. The putative targets of the miRNAs were determined using the IPA microRNA Target Filter. We performed a Core Pathway Analysis using miRNA targets to identify relationships among the mRNAs in our dataset. The canonical pathways with *p*-value < 0.05 (Fischer’s exact test) were considered statistically significant, and the activation z-score was calculated to predict the activation or inhibition of transcriptional regulators (z-score ≠ 0) based on published findings accessible through IPA, as previously described in [28].

## 3. Results

### 3.1. Circulating Total EV microRNA Expression Is Altered by Aging

**Figure 1:**
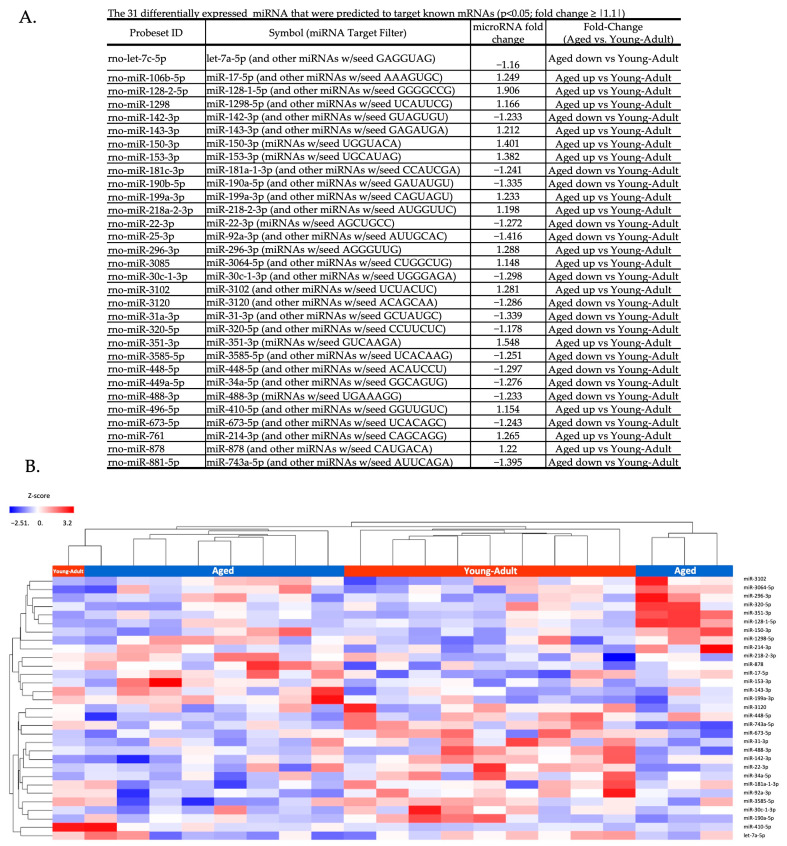
Differentially expressed mature miRNA. (**A**). The 31 mature miRNAs with targets, based on current available IPA target annotations. The miRNA fold-change between aged and young adult rats is summarized. (**B**) Hierarchical clustering of the expression of differently expressed miRNAs from aged and young adult rats. The color bar indicates the standardized expression (z-score) of each miRNA to a mean of 0. Upregulated microRNAs have positive values and are displayed in red. Downregulated microRNAs have negative values and are displayed in blue.

The analysis of microRNA expression showed 728 microRNAs present in the circulating EVPs. Of the detected circulating EVP microRNAs, 48 were differentially expressed between the aged and young adult groups (*p* < 0.05; fold change ≥ |1.1|); 18 were upregulated and 30 were downregulated in the aged group compared to young adult animals (Supplementary Table S1). The predicted effects of age-related microRNA differences on target mRNAs were investigated using QIAGEN Ingenuity Pathway Analysis (IPA). Of the 48 differentially expressed microRNAs, 31 mature microRNAs had identifiable target information available based on current annotations (Figure 1a). The unsupervised hierarchical cluster heatmap shows the relative intensity of differential expression for these 31 microRNAs (Figure 1b). 

We found that these 31 mature microRNAs putatively target 5901 mRNAs; 272 canonical pathways were identified as significantly up- or down-regulated in the aged compared to the young adult group (*p* < 0.05 and z-score (a measure of pathway activation/inhibition) ≠ 0) [29]. Supplementary Table S2 ranks the canonical pathways according to the value of the −log *p*-value. 

### 3.2. Cardiovascular Signaling and Redox Pathways Are Predicted to Be Impacted by Circulating Total EV microRNA from Aged Animals

**Figure 2:**
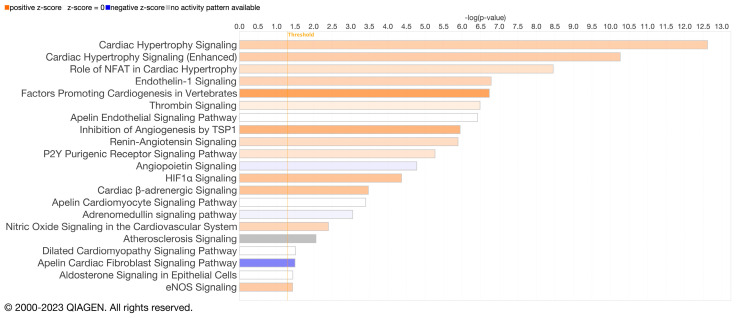
Cardiovascular signaling canonical pathways. The top cardiovascular signaling canonical pathways with predicted targets for differentially expressed total EVP microRNAs between aged and young animals were identified by Ingenuity pathway analysis (IPA). Canonical pathways are displayed along the y-axis and the −log (*p*-value), calculated by Fisher’s exact test right-tailed, on the x-axis. The threshold line indicates the minimum significance level −log (*p*-value). The length of the bars represents significance, with longer bars representing more significant associations, and the orange color indicates that those pathways are predicted to be upregulated (z-score >0), while blue indicates downregulation (z-score < 0).

**Figure 3:**
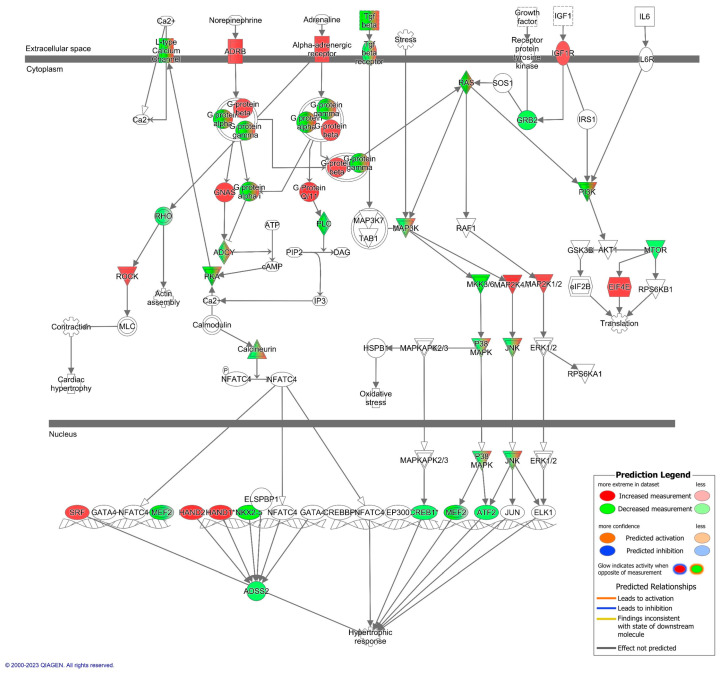
The Cardiac Hypertrophy Signaling pathway is predicted to be upregulated in EVPs from aged animals. Predicted up- and downregulation of mRNA targets in the cardiac hypertrophy signaling pathway. The red color indicates predicted upregulation while the green indicates predicted downregulation. The complete list of pathway targets and their predicted fold-change can be found in Supplementary Table S3.

Cardiac hypertrophy signaling (Supplementary Table S2; Figure 2 and Figure 3) was ranked as the top canonical pathway (based on *p*-values) impacted by microRNAs that were differentially expressed between the aged and young groups. Given that cardiac hypertrophy signaling was the top pathway, and that cardiovascular diseases are the leading cause of death worldwide, we focused our attention on cardiovascular signaling pathways and the mechanisms that might contribute to cardiovascular disease. 

Figure 2 presents the top cardiovascular signaling canonical pathways predicted to be affected by differentially expressed EVP microRNAs between aged and young animals. Approximately 61% of the mapped microRNAs (19 of 31 microRNA) were predicted to target molecules involved in cardiac hypertrophy during aging (Supplementary Table S3). Figure 3 highlights important components of the cardiac hypertrophic signaling pathway, such as β-adrenergic receptors. Different microRNAs that target β-adrenergic receptors (β-AR)—including let-7a-5p, miR-34a-5p, and miR-92a-3p—were also impacted by aging. Additionally, the cAMP-dependent protein kinase A (PKA)—a key enzyme for cardiac function [30]—appears to be regulated by let-7a-5p and miR-128-1-5p. 

**Figure 4:**
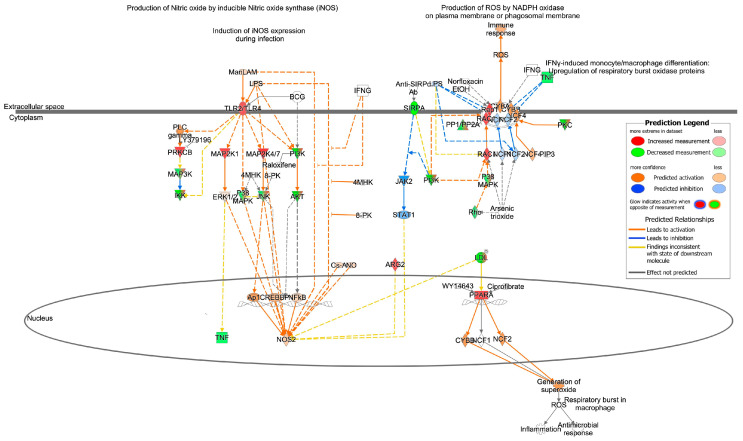
Production of Nitric Oxide and Reactive Oxygen Species in Macrophages signaling pathways is predicted to be upregulated in EVPs from aged animals. Predicted up- and downregulation of mRNA targets in the cardiac hypertrophy signaling pathway. The red color indicates predicted upregulation while the green indicates predicted downregulation. The complete list of pathway targets and their predicted fold-change can be found in Supplementary Table S5.

Since oxidative stress mechanisms have been widely linked to cardiovascular diseases and the development of cardiac hypertrophy, we also examined predicted changes in pathways related to oxidative stress. The effects of the age-related EVP miRNA profile on oxidative stress signaling pathways were investigated using IPA. The canonical pathways predicted to be modified in aged animals when compared to the young adult group are shown in Supplementary Table S4. IPA analysis showed that Production of Nitric Oxide and Reactive Oxygen Species in Macrophages (*p* < 0.001, z-score= 0.412) and Nitric Oxide Signaling in the Cardiovascular System (*p* = 0.004, z-score = +1.043) are canonical pathways predicted to be upregulated by differentially expressed miRNAs in the aging process (Supplementary Table S4). We observed that 19 miRNAs putatively target 56 molecules in the Production of Nitric Oxide and Reactive Oxygen Species in the Macrophages signaling pathway (Supplementary Table S5), while in the nitric oxide signaling in the cardiovascular system pathway, 15 miRNAs target 31 molecules. Figure 4 highlights the important targets of microRNAs within the Nitric Oxide and Reactive Oxygen Species in Macrophages. The rho-like small GTPase, RAC1, has a critical role in NADPH oxidase activation [31] and is predicted to be upregulated by miR-142-3p in the pathway Production of Nitric Oxide and Reactive Oxygen Species in Macrophages. Moreover, miR-22-3p is downregulated in our dataset and targets peroxisome proliferator-activated receptor alpha (PPAR-α)—this results in a predicted upregulation of PPAR-α, which in turn is predicted to activate the CYBB and NCF2 subunits of NADPH oxidase.

### 3.3. Redox Machinery in EVPs

**Figure 5:**
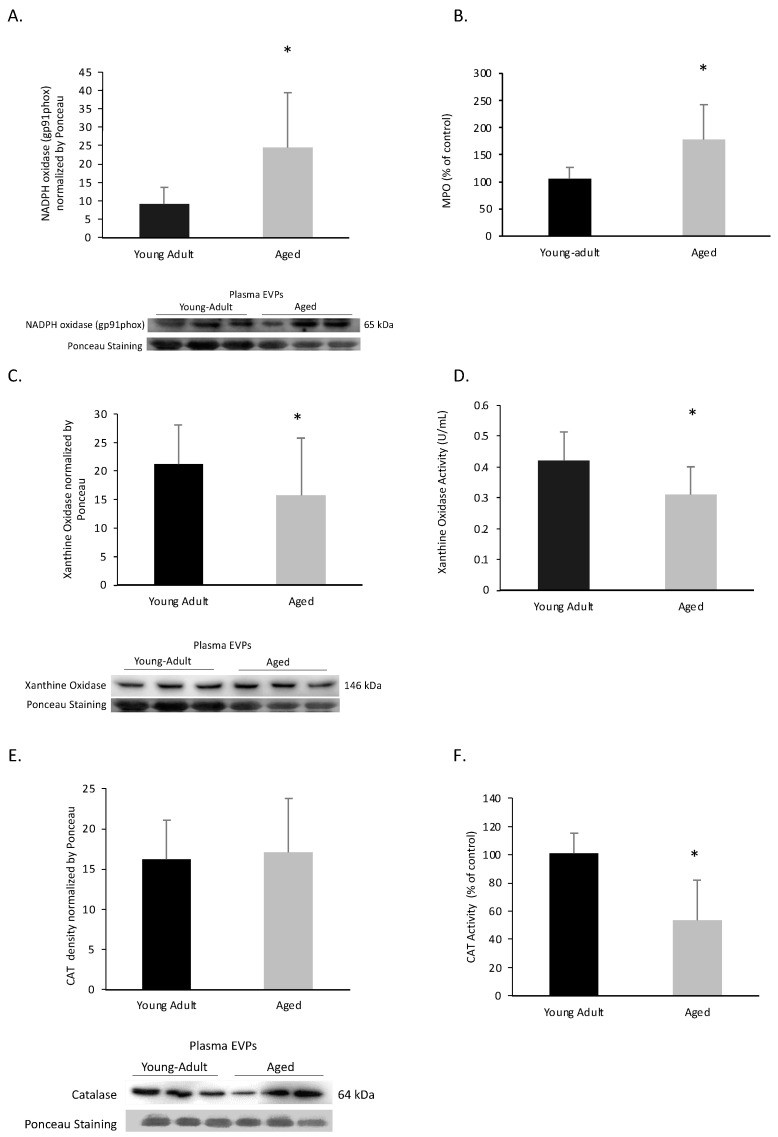
Effect of the aging process on pro- and antioxidant markers in circulating extracellular vesicles and particles (EVPs) from Wistar rats, of 3- and 21-month-old. Columns represent the mean ± SD; representative images from a gel of Western Blot experiments, showing 3 bands for each experimental group. Student’s *t*-test; * significantly different from young adults, *p* < 0.05. (**A**) NADPH oxidase content (*n* = 6; *p* = 0.043); (**B**) MPO activity (*n* = 9; *p* = 0.008); (**C**) xanthine oxidase content (*n* = 6; *p* = 0.040); and (**D**) xanthine oxidase activity (*n* = 9; *p* = 0.024); (**E**) catalase content (*n* = 6; *p* = 0.34) and (**F**) catalase activity (*n* = 9; 0.0002).

To validate some of the findings predicted by the IPA, we evaluated the content and activity of redox enzymes. Circulating total EVPs obtained from aged rats had significantly higher NADPH oxidase levels, as assessed by gp91phox subunit content (also known as NOX2), when compared to young adult rats (Student’s *t*-test; *p* = 0.043, Figure 5A). In addition, aged rats showed significantly higher MPO activity compared to young adults (*p* = 0.008, Figure 5B). A lower content and activity of XO were observed in total EVPs obtained from aged rats compared to young adult rats (Student’s *t*-test; respectively, *p* = 0.040, Figure 5C; *p* = 0.024, Figure 5D). 

EVPs obtained from 21-month-old rats had reduced CAT activity (Student’s *t*-test; *p* = 0.0002, Figure 5F). However, there was no significant difference in CAT content between the tested age groups (*p* = 0.34, Figure 5E). 

### 3.4. Characterization of Isolated EVPs

Our characterization analysis showed that neither FLOT1—a known EVP marker [32,33]—nor EVP size and concentration were statistically significant between young adult and aged rats (Student’s *t*-test; *p* = 0.485, *p* = 0.698, *p* = 0.407, Supplementary Figure S1). 

## 4. Discussion

The data presented here support the hypothesis that aging impacts the miRNA profiles of circulating EVPs. In this study, we identified predicted targets, canonical pathways, and cellular mechanisms influenced by the age-induced differential expression of miRNAs in EVPs. Moreover, EVPs cargo seems to be altered by the aging process.

We showed here, for the first time, that EVPs can be a potential mechanism of spreading molecules involved in susceptibility to chronic diseases afflicting the elderly. Our data show that the altered miRNA profile and redox content of EVPs contribute to the permissive environment that is responsible, at least in part, for age-associated diseases and geriatric syndromes.

Cardiovascular disease is the number one cause of death worldwide and the elderly are the most susceptible [17]. Interestingly, cardiac hypertrophy signaling was the top canonical pathway impacted by differentially expressed miRNAs in circulating total EVPs from aged rats. Even in healthy individuals without any cardiovascular disease, the prevalence of left ventricular hypertrophy is increased with age—independent of any predictable risk factors [34,35,36]—and thus, cardiac hypertrophy is considered a hallmark of cardiac aging [35]. Previous data have described several miRNAs in cardiac tissue associated with cardiomyocyte hypertrophic signaling, both in human and rodent models of hypertrophic cardiomyopathy—including miR-214 [37], miR-199a [38], and let-7 [39]. Although the involvement of miRNA expression in hypertrophic cardiomyopathy has previously been suggested [40], to our knowledge, the circulating total EVP miRNA profile has been poorly explored in aged animals. We showed here that the EVP miRNA profile in aged animals can be related to cardiac hypertrophy signaling. 

Cardiac hypertrophy signaling has previously been associated with miR-34a [41], which we found to be differentially expressed between the aged and young adults’ circulating total EVP miRNA. In addition, our data showed that miR-34a-5p, let-7a-5p, and miR-92a-3p—which are downregulated in the EVPs of aged animals compared to young—are predicted to target cardiac adrenergic receptors, which may play a crucial role in the development of cardiovascular dysfunction observed with advanced age and its progression to heart failure [42]. The stimulation of adrenergic receptors triggers the activation of G-proteins; however, we observed an intriguing alteration in the activity pattern of G protein subunits gamma (Gγ), alpha (Gα), and beta (Gβ) that arises from the deregulated miRNAs—including let-7a-5p, miR-34a-5p, and miR-92a-3p. Additional research is required to elucidate this matter. Moreover, a key enzyme for cardiac function—cAMP-dependent protein kinase A (PKA, specifically PRKAR2A) [30]—is predicted to be upregulated by let-7a-5p in circulating total EVPs during the aging process. Interestingly, Enns et al. [43] suggested that reduced PKA function mediates anti-aging effects, demonstrating that wild C57BL/6 mice have continually increased cardiac dysfunction as early as 10–12 months of age, while male mutant mice lacking the PKA regulatory IIα subunit (PRKAR2A) have delayed cardiac decline and an increased lifespan [43]. 

In addition to changes in adrenergic receptors and other molecules such as G-proteins and PKA, there is a well-established association between oxidative stress mechanisms and cardiovascular diseases, including the development of cardiac hypertrophy. Our study revealed that the differential expression of miRNAs in circulating total EVPs has the potential to impact oxidative stress signaling pathways and predict an upregulation of several cardiac-related pathways. Engedal and colleagues [18] reviewed the published findings on oxidative stress-dependent miRNA expression and, using in silico tools, identified several oxidative stress-modulated miRNAs, their predicted mRNA gene targets, and the affected canonical pathways; these authors described that 13 miRNAs—let-7, miR-9, miR-16, miR-21, miR-22, miR-29b, miR-99a, miR-125b, miR-128, miR-143, miR-144, miR-155, and miR-200c—seem to be modulated by oxidative stress [18]. Our analysis in IPA identified targets in the Production of Nitric Oxide and Reactive Oxygen Species in Macrophages pathway—among them, RAC1 was a predicted target of miR-142-3p. It has already been reported that RAC1 acts through NAPDH oxidase activation to induce myocardial remodeling and dysfunction. Li and colleagues [44] showed in a model of type 1 diabetic mice that NADPH oxidase activation and expression induce ROS production and inflammation, and this is attenuated by RAC1 knockout [44]. Moreover, Wang et al. [45] reported that miR-142-3p inhibits the apoptosis and fibrosis of cardiomyocytes—in part by the direct inhibition of HMGB1 expression [45]. Given the data presented here, we propose that a reduction in circulating EVP miR-142-3p levels during aging might indicate a loss of a protective mechanism against cardiomyocyte fibrosis and cell death.

Other main targets in the Production of Nitric Oxide and Reactive Oxygen Species in Macrophages pathway are phosphatases, which are known to control diverse cellular processes including kinase cascades, cell growth, and apoptosis [46]. PPP1R7 and PPP2R2A are predicted to be regulated by let-7a-5p and miR-199a-3p, which are differentially expressed in our study in aged vs. young rats. Besides this, Rho family small GTPases (RHOB, RHOG), predicted to be upregulated by let-7a-5p, are essential for the activation of the NOX family in animals [47]. Additionally, PPAR-α was also reported as a target of differentially expressed EVP miRNA—namely, miR-22-3p. Prior work has shown that increased PPAR-α expression is observed in human dilated cardiomyopathy [48]. Moreover, PPAR-α activation can induce ROS production in macrophages via the NADPH oxidase pathway [49], which may have implications for cardiac hypertrophy. 

As the aging process can affect not only the miRNAs, but also the redox machinery in circulating total EVPs—leading to an imbalance between pro- and antioxidant systems—our work examined these enzymes in EVPs. We investigated the levels of redox enzymes found in EVPs that were also linked to physiological aging processes and cardiovascular disease. Bertoldi and colleagues [2] previously found that the levels of reactive species are increased in circulating EVPs from aged rats compared to young rats; however, no work has previously demonstrated an increased level of reactive species-producing machinery in total EVPs. Here, we have demonstrated increased NADPH oxidate levels in total EVPs, which may explain the increased level of reactive species. However, we cannot exclude oxidative stress in tissues and organs as a circulating reactive species source in EVPs. EVP intrinsic production, specifically by elevated NADPH oxidase, seems to play a central role in age-related oxidative status. Moreover, there is evidence supporting that EVPs can deliver NADPH oxidase from parental to target cells; specifically, exosomes derived from macrophages containing functional NADPH oxidase were previously shown to be incorporated in axons via endocytosis, affecting axonal signaling [50].

Different approaches have found that MPO can be cargo in polymorphonuclear (PMN) cell-derived extracellular vesicles. Pitanga and colleagues [51] showed that PMN-derived EVs are able to produce reactive oxygen species and proinflammatory mediators such as leukotriene B4. Considering that both NADPH oxidase and MPO are involved in the production of reactive oxygen species in PMN cells, through a process called respiratory burst (or oxidative burst); it is possible to infer the role of circulating EVPs in spreading respiratory burst machinery in the aging process.

While Aranda and colleagues [52] found that XO expression and activity are increased in the plasma of elderly humans and in the plasma, skeletal muscle, and aorta of aged rats, our results demonstrated reduced XO activity and content in circulating EVPs from aged rats; this suggests that there may be a decreased loading of XO into circulating EVPs and may indicate a failure of cellular waste disposal processes [53,54].

To better comprehend the impact of the aging process on the redox machinery in circulating EVPs, antioxidant defenses were evaluated as well. It was previously reported that the superoxide dismutase (SOD) content and activity were reduced in circulating total EVPs from 26-month-old rats [2]. Lower CAT activity in circulating EVPs from aged animals is another remarkable finding; we can infer a decreased exchange of protective molecules from cells to EVPs. A previous study evaluated the neuroprotective potential of EVPs from mesenchymal stem cells using an in vitro model of Alzheimer’s disease and reported that mesenchymal stem cell-derived EVPs were able to reduce reactive oxygen species levels in neuronal cell cultures [55]. It is possible that our result reflects the tissue CAT activity [8,56]. Therefore, we suggest that impaired antioxidant enzyme activity contributes to global oxidative stress in older animals. 

Our findings concerning the potential role of the circulating total EVP miRNA signature for age-related phenotypes can support those that have been previously reported. EVPs might be the circulating factors described in a study performed by Loffredo and colleagues [57] using heterochronic parabiosis, where circulating factors seem to be involved in aging-induced pathological cardiac hypertrophy [57]. In addition, 26-month-old mice had an increased lifespan after receiving circulating total EVPs isolated from young animals [58]. In addition, Lee and colleagues [16] showed that the administration of exosomes obtained from young mice altered the expression pattern of aging-associated molecules in aged mice [16]. 

## 5. Conclusions

Our data concerning the miRNA profile in circulating total EVPs indicates they may play a role in age-related phenotypes—especially cardiac hypertrophy. In addition, we provide evidence that the aging process can impair pro-oxidant and antioxidant molecules in circulating total EVPs from aged animals. Our results give insight into the role of the circulating EVP signature, specifically miRNAs and redox enzymes, in the aging process and open new avenues for further studies. 

## Data Availability

The data presented in this study are openly available in NCBI Gene Expression Omnibus (GEO Accession number: GSE243656).

## Supplementary Materials

The following supporting information can be downloaded at: https://www.mdpi.com/article/10.3390/biomedicines11112920/s1, Supplementary Table S1. The 48 differentially expressed microRNA between the aged and young adult groups (*p* < 0.05; fold change ≥ |1.1|) observed in Partek Analysis. Supplementary Table S2. Canonical Pathways predicted to be altered by circulating total EVP miRNAs. Pathways are listed according to their value of −log *p*-value and predicted z-score (measure of pathway activation/inhibition) ≠ 0. Supplementary Table S3. Cardiac Hypertrophy Signaling pathway predicted targets. Supplementary Table S4. List of oxidative stress-related canonical pathways predicted to be targeted by differentially expressed miRNAs in circulating total EVPs. Supplementary Table S5. Production of Nitric Oxide and Reactive Oxygen Species Macrophages Canonical Pathway predicted targets. Supplementary Figure S1. EVPs characterization.
