# Supplementary material for: Circulating Total Extracellular Vesicles Cargo Are Associated with Age-Related Oxidative Stress and Susceptibility to Cardiovascular Diseases: Exploratory Results from Microarray Data
Source: Biomedicines. 2023 Oct 28;11(11):2920. doi: 10.3390/biomedicines11112920 (PMC10669226; doi:10.3390/biomedicines11112920)
Supplement: Supplementary file 1 [file biomedicines-11-02920-s001.zip › Supplementary Figure S1.pdf]

Supplementary Figure S1.

A.

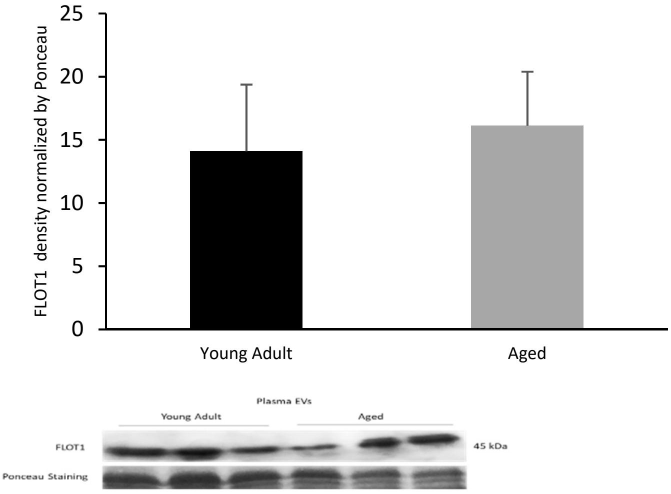

B.

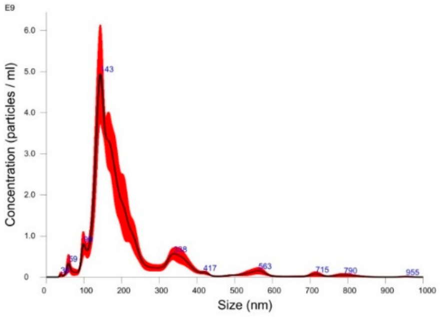

C.

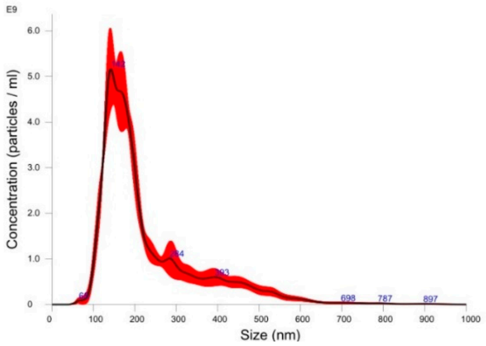

D.

	NTA characterization of extracellular vesicles	
	Mean Diameter (nm)	Concentration (particles/mL)
**Young Adult**	223.11 ± 28.62	$1.07 \times 10^{12} \pm 5.07 \times 10^{11}$
**Aged**	218.5 ± 23.37	$1.36 \times 10^{12} \pm 8.50 \times 10^{11}$

NTA data on EVs size distribution and concentration from Wistar rats, of 3- and 21-month-old. Student's t-test; $p<0.05$ (n=9/group).

Supplementary Figure S1. (A) Effect of aging process on **FLOT1 content** in circulating extracellular vesicles from *Wistar* rats of 3- and 21-month-olds. The columns represent the mean ± SD. Representative images from

the gel of Western Blot experiments, showing 3 bands for each experimental group. Student's t-test; $p<0.05$ (n=6). Representative data of **NTA analysis** showing size distribution profile and concentration of circulating EVs from **(B)** young-adult (3-month-old) and **(C)** aged (21-month-old) *Wistar* rats (n=9) **(D).** NTA data on EVP size distribution and concentration from Wistar rats of 3- and 21-month-olds. Student's t-test; $p<0.05$ (n=9/group).
