# Supplementary material for: Circulating Total Extracellular Vesicles Cargo Are Associated with Age-Related Oxidative Stress and Susceptibility to Cardiovascular Diseases: Exploratory Results from Microarray Data
Source: Biomedicines. 2023 Oct 28;11(11):2920. doi: 10.3390/biomedicines11112920 (PMC10669226; doi:10.3390/biomedicines11112920)
Supplement: Supplementary file 1 [file biomedicines-11-02920-s001.zip › Supplementary Table S1.pdf]

Supplementary Table S1

The 48 differentially expressed microRNA between the aged and young-adult groups (p<0.05; fold change ≥ |1.1|) observed in Partek Analysis.

Probeset ID	Fold-Change (Aged vs. Young-Adult)	Fold-Change (Aged vs. Young-Adult)
rno-let-7c-5p	-1.16028	Aged down vs Young-Adult
rno-miR-106b-5p	1.24874	Aged up vs Young-Adult
rno-miR-128-2-5p	1.90585	Aged up vs Young-Adult
rno-miR-1298	1.16639	Aged up vs Young-Adult
rno-miR-142-3p	-1.23301	Aged down vs Young-Adult
rno-miR-143-3p	1.21171	Aged up vs Young-Adult
rno-miR-150-3p	1.40147	Aged up vs Young-Adult
rno-miR-153-3p	1.38177	Aged up vs Young-Adult
rno-miR-181a-1-3p	-1.2052	Aged down vs Young-Adult
rno-miR-181c-3p	-1.24133	Aged down vs Young-Adult
rno-miR-190b-5p	-1.33542	Aged down vs Young-Adult
rno-miR-199a-3p	1.23322	Aged up vs Young-Adult
rno-miR-214-5p	-1.30019	Aged down vs Young-Adult
rno-miR-218a-2-3p	1.19794	Aged up vs Young-Adult
rno-miR-22-3p	-1.27173	Aged down vs Young-Adult
rno-miR-25-3p	-1.41609	Aged down vs Young-Adult
rno-miR-296-3p	1.28763	Aged up vs Young-Adult
rno-miR-3085	1.14837	Aged up vs Young-Adult
rno-miR-30c-1-3p	-1.29773	Aged down vs Young-Adult
rno-miR-3102	1.28118	Aged up vs Young-Adult
rno-miR-3120	-1.28576	Aged down vs Young-Adult
rno-miR-31a-3p	-1.33894	Aged down vs Young-Adult
rno-miR-31b	-1.25732	Aged down vs Young-Adult
rno-miR-320-5p	-1.17829	Aged down vs Young-Adult
rno-miR-34b-5p	-1.26043	Aged down vs Young-Adult
rno-miR-351-3p	1.54788	Aged up vs Young-Adult
rno-miR-3559-5p	-1.28751	Aged down vs Young-Adult
rno-miR-3580-3p	-1.35608	Aged down vs Young-Adult
rno-miR-3580-5p	-1.23411	Aged down vs Young-Adult
rno-miR-3583-5p	-1.25473	Aged down vs Young-Adult
rno-miR-3585-5p	-1.25096	Aged down vs Young-Adult
rno-miR-3590-5p	-1.2942	Aged down vs Young-Adult
rno-miR-379-3p	-1.24993	Aged down vs Young-Adult
rno-miR-409b	1.20213	Aged up vs Young-Adult
rno-miR-421-5p	-1.17373	Aged down vs Young-Adult
rno-miR-448-5p	-1.29741	Aged down vs Young-Adult
rno-miR-449a-5p	-1.27571	Aged down vs Young-Adult

rno-miR-451-3p	-1.21042	Aged down vs Young-Adult
rno-miR-488-3p	-1.23306	Aged down vs Young-Adult
rno-miR-496-5p	1.1538	Aged up vs Young-Adult
rno-miR-6330	-1.3321	Aged down vs Young-Adult
rno-miR-6332	1.31066	Aged up vs Young-Adult
rno-miR-673-5p	-1.24319	Aged down vs Young-Adult
rno-miR-743a-3p	-1.22295	Aged down vs Young-Adult
rno-miR-761	1.26548	Aged up vs Young-Adult
rno-miR-878	1.21965	Aged up vs Young-Adult
rno-miR-881-5p	-1.39481	Aged down vs Young-Adult
rno-miR-92a-3p	1.19759	Aged up vs Young-Adult
