# Supplementary material for: Circulating Total Extracellular Vesicles Cargo Are Associated with Age-Related Oxidative Stress and Susceptibility to Cardiovascular Diseases: Exploratory Results from Microarray Data
Source: Biomedicines. 2023 Oct 28;11(11):2920. doi: 10.3390/biomedicines11112920 (PMC10669226; doi:10.3390/biomedicines11112920)
Supplement: Supplementary file 1 [file biomedicines-11-02920-s001.zip › Supplementary Table S2.pdf]

Supplementary Table S2.

| Ingenuity Canonical Pathways | -log(p-value) | p-value | z-score |
|---|---|---|---|
| Cardiac Hypertrophy Signaling | 12.6 | 2.51E-13 | 1.195 |
| CCR3 Signaling in Eosinophils | 11.2 | 6.31E-12 | 1.183 |
| Colorectal Cancer Metastasis Signaling | 10.7 | 2E-11 | 2.425 |
| Cardiac Hypertrophy Signaling (Enhanced) | 10.3 | 5.01E-11 | 1.237 |
| Pulmonary Fibrosis Idiopathic Signaling Pathway | 10.3 | 5.01E-11 | 3.637 |
| Opioid Signaling Pathway | 10.1 | 7.94E-11 | -0.465 |
| Hepatic Fibrosis Signaling Pathway | 10.1 | 7.94E-11 | 2.44 |
| Senescence Pathway | 9.72 | 1.91E-10 | -1 |
| Glioblastoma Multiforme Signaling | 9.49 | 3.24E-10 | 1.697 |
| MYC Mediated Apoptosis Signaling | 9.31 | 4.9E-10 | 1.732 |
| G-Protein Coupled Receptor Signaling | 9.26 | 5.5E-10 | 1.436 |
| CREB Signaling in Neurons | 9.1 | 7.94E-10 | 2.629 |
| Epithelial Adherens Junction Signaling | 8.98 | 1.05E-09 | 0.927 |
| Glioma Signaling | 8.5 | 3.16E-09 | 1.134 |
| Role of NFAT in Cardiac Hypertrophy | 8.45 | 3.55E-09 | 0.762 |
| Endocannabinoid Developing Neuron Pathway | 8.45 | 3.55E-09 | 0.949 |
| Neuroinflammation Signaling Pathway | 8.33 | 4.68E-09 | 1.565 |
| GNRH Signaling | 8.32 | 4.79E-09 | 0.566 |
| Endocannabinoid Cancer Inhibition Pathway | 8.2 | 6.31E-09 | -0.849 |
| ERBB Signaling | 8.14 | 7.24E-09 | 0.493 |
| PEDF Signaling | 8.07 | 8.51E-09 | 0.845 |
| IL-8 Signaling | 7.92 | 1.2E-08 | 2.252 |
| CLEAR Signaling Pathway | 7.88 | 1.32E-08 | -2.182 |

| | | | |
|---|---|---|---|
| Cholecystokinin/Gastrin-mediated Signaling | 7.7 | 2E-08 | 0.156 |
| NGF Signaling | 7.66 | 2.19E-08 | 0.617 |
| Ovarian Cancer Signaling | 7.26 | 5.5E-08 | 1 |
| Pancreatic Adenocarcinoma Signaling | 7.12 | 7.59E-08 | 0.898 |
| Pulmonary Healing Signaling Pathway | 7.01 | 9.77E-08 | 2.032 |
| Breast Cancer Regulation by Stathmin1 | 6.93 | 1.17E-07 | 1.781 |
| Tumor Microenvironment Pathway | 6.86 | 1.38E-07 | 0.927 |
| CXCR4 Signaling | 6.85 | 1.41E-07 | 1.809 |
| FAK Signaling | 6.85 | 1.41E-07 | 2.49 |
| p38 MAPK Signaling | 6.84 | 1.45E-07 | 0.48 |
| Acute Myeloid Leukemia Signaling | 6.8 | 1.58E-07 | 0.365 |
| Endothelin-1 Signaling | 6.78 | 1.66E-07 | 1.069 |
| Factors Promoting Cardiogenesis in Vertebrates | 6.73 | 1.86E-07 | 2.263 |
| Oxytocin In Brain Signaling Pathway | 6.6 | 2.51E-07 | 0.651 |
| PTEN Signaling | 6.52 | 3.02E-07 | -0.617 |
| Regulation Of The Epithelial Mesenchymal Transition By Growth Factors Pathway | 6.52 | 3.02E-07 | 0.272 |
| Thrombin Signaling | 6.48 | 3.31E-07 | 0.42 |
| Apoptosis Signaling | 6.44 | 3.63E-07 | 0.493 |
| Renal Cell Carcinoma Signaling | 6.34 | 4.57E-07 | 0.626 |
| Oxytocin Signaling Pathway | 6.31 | 4.9E-07 | 0.798 |
| ERK/MAPK Signaling | 6.31 | 4.9E-07 | 1.483 |
| SAPK/JNK Signaling | 6.26 | 5.5E-07 | 1.333 |
| Non-Small Cell Lung Cancer Signaling | 6.24 | 5.75E-07 | 1.043 |
| 14-3-3-mediated Signaling | 6.22 | 6.03E-07 | 0.667 |
| Gαq Signaling | 6.16 | 6.92E-07 | 1.809 |
| CDK5 Signaling | 6.14 | 7.24E-07 | 0.667 |
| Role Of Osteoclasts In Rheumatoid Arthritis Signaling Pathway | 6.12 | 7.59E-07 | 2.621 |

| | | | |
|---|---|---|---|
| LPS-stimulated MAPK Signaling | 6.08 | 8.32E-07 | 1.616 |
| Inhibition of Angiogenesis by TSP1 | 5.95 | 1.12E-06 | 1.807 |
| Production of Nitric Oxide and Reactive Oxygen Species in Macrophages | 5.92 | 1.2E-06 | 0.412 |
| Endometrial Cancer Signaling | 5.92 | 1.2E-06 | 1.606 |
| Melanoma Signaling | 5.9 | 1.26E-06 | 0.655 |
| Renin-Angiotensin Signaling | 5.89 | 1.29E-06 | 0.845 |
| STAT3 Pathway | 5.78 | 1.66E-06 | 1.808 |
| TGF-β Signaling | 5.75 | 1.78E-06 | 1.347 |
| Systemic Lupus Erythematosus In T Cell Signaling Pathway | 5.72 | 1.91E-06 | 0.75 |
| Gαi Signaling | 5.58 | 2.63E-06 | 0.507 |
| B Cell Receptor Signaling | 5.47 | 3.39E-06 | -0.412 |
| ERB2-ERBB3 Signaling | 5.43 | 3.72E-06 | 0.6 |
| Small Cell Lung Cancer Signaling | 5.38 | 4.17E-06 | 0.471 |
| CDX Gastrointestinal Cancer Signaling Pathway | 5.38 | 4.17E-06 | 1.313 |
| Cell Cycle: G1/S Checkpoint Regulation | 5.37 | 4.27E-06 | -2.294 |
| HGF Signaling | 5.37 | 4.27E-06 | 1.298 |
| PDGF Signaling | 5.31 | 4.9E-06 | 1.616 |
| P2Y Purigenic Receptor Signaling Pathway | 5.27 | 5.37E-06 | 0.667 |
| Role of NANOG in Mammalian Embryonic Stem Cell Pluripotency | 5.25 | 5.62E-06 | 0.655 |
| ID1 Signaling Pathway | 5.15 | 7.08E-06 | -0.263 |
| Mouse Embryonic Stem Cell Pluripotency | 5.14 | 7.24E-06 | 0.686 |
| Thyroid Cancer Signaling | 5.14 | 7.24E-06 | 1.671 |
| T Cell Exhaustion Signaling Pathway | 5.03 | 9.33E-06 | -0.174 |
| Reelin Signaling in Neurons | 5 | 0.00001 | 0.617 |
| FLT3 Signaling in Hematopoietic Progenitor Cells | 4.98 | 1.05E-05 | 0.557 |
| p70S6K Signaling | 4.97 | 1.07E-05 | 0.649 |
| Fc Epsilon RI Signaling | 4.93 | 1.17E-05 | 1.521 |

| | | | |
|---|---|---|---|
| Phagosome Formation | 4.91 | 1.23E-05 | 2.959 |
| IL-3 Signaling | 4.88 | 1.32E-05 | 1.134 |
| Endocannabinoid Neuronal Synapse Pathway | 4.86 | 1.38E-05 | -0.156 |
| IGF-1 Signaling | 4.82 | 1.51E-05 | 0.928 |
| Integrin Signaling | 4.81 | 1.55E-05 | 1.483 |
| Dopamine-DARPP32 Feedback in cAMP Signaling | 4.78 | 1.66E-05 | -1.265 |
| Angiopoietin Signaling | 4.78 | 1.66E-05 | -0.218 |
| Neurotrophin/TRK Signaling | 4.78 | 1.66E-05 | 0.408 |
| Macropinocytosis Signaling | 4.78 | 1.66E-05 | 2.236 |
| Chemokine Signaling | 4.78 | 1.66E-05 | 2.502 |
| WNT/β-catenin Signaling | 4.7 | 2E-05 | 1.508 |
| MicroRNA Biogenesis Signaling Pathway | 4.65 | 2.24E-05 | 1.54 |
| BMP signaling pathway | 4.62 | 2.4E-05 | 1.134 |
| Synaptogenesis Signaling Pathway | 4.61 | 2.45E-05 | -0.465 |
| Ceramide Signaling | 4.61 | 2.45E-05 | 0.186 |
| Necroptosis Signaling Pathway | 4.56 | 2.75E-05 | 0.302 |
| Natural Killer Cell Signaling | 4.55 | 2.82E-05 | -0.849 |
| HMGB1 Signaling | 4.55 | 2.82E-05 | 0.949 |
| Sphingosine-1-phosphate Signaling | 4.53 | 2.95E-05 | 0.174 |
| Systemic Lupus Erythematosus In B Cell Signaling Pathway | 4.5 | 3.16E-05 | -0.12 |
| G Beta Gamma Signaling | 4.48 | 3.31E-05 | 0.973 |
| GM-CSF Signaling | 4.44 | 3.63E-05 | 0.853 |
| AMPK Signaling | 4.42 | 3.8E-05 | -0.309 |
| TEC Kinase Signaling | 4.42 | 3.8E-05 | 0.686 |
| BEX2 Signaling Pathway | 4.41 | 3.89E-05 | 0.192 |
| Semaphorin Neuronal Repulsive Signaling Pathway | 4.39 | 4.07E-05 | -1.067 |
| FGF Signaling | 4.39 | 4.07E-05 | 0.73 |

| | | | |
|---|---|---|---|
| HIF1α Signaling | 4.37 | 4.27E-05 | 1.483 |
| Agrin Interactions at Neuromuscular Junction | 4.34 | 4.57E-05 | -0.426 |
| IL-7 Signaling Pathway | 4.32 | 4.79E-05 | 0.218 |
| GDNF Family Ligand-Receptor Interactions | 4.3 | 5.01E-05 | 1.633 |
| UVA-Induced MAPK Signaling | 4.29 | 5.13E-05 | 0.471 |
| VEGF Family Ligand-Receptor Interactions | 4.29 | 5.13E-05 | 1.347 |
| Cyclins and Cell Cycle Regulation | 4.28 | 5.25E-05 | 0.853 |
| Actin Nucleation by ARP-WASP Complex | 4.28 | 5.25E-05 | 2.711 |
| PCP (Planar Cell Polarity) Pathway | 4.27 | 5.37E-05 | 2.132 |
| PI3K/AKT Signaling | 4.2 | 6.31E-05 | 0.156 |
| IL-1 Signaling | 4.19 | 6.46E-05 | 0.243 |
| Protein Kinase A Signaling | 4.14 | 7.24E-05 | 0.226 |
| PAK Signaling | 4.14 | 7.24E-05 | 0.898 |
| Synaptic Long Term Potentiation | 4.11 | 7.76E-05 | -0.649 |
| Role of MAPK Signaling in Promoting the Pathogenesis of Influenza | 4.11 | 7.76E-05 | 1.567 |
| IL-13 Signaling Pathway | 4.09 | 8.13E-05 | 0.354 |
| IL-17 Signaling | 4.06 | 8.71E-05 | -0.295 |
| PKCθ Signaling in T Lymphocytes | 4.03 | 9.33E-05 | 0.169 |
| mTOR Signaling | 4.01 | 9.77E-05 | 3.042 |
| Huntington's Disease Signaling | 3.97 | 0.000107 | 0.457 |
| Insulin Secretion Signaling Pathway | 3.95 | 0.000112 | -1.069 |
| fMLP Signaling in Neutrophils | 3.95 | 0.000112 | 1.616 |
| Bladder Cancer Signaling | 3.94 | 0.000115 | 1.291 |
| Estrogen-mediated S-phase Entry | 3.93 | 0.000117 | 1.941 |
| Cell Cycle Regulation by BTG Family Proteins | 3.92 | 0.00012 | 0.632 |
| Telomerase Signaling | 3.91 | 0.000123 | 0.408 |
| GADD45 Signaling | 3.9 | 0.000126 | 0.426 |

| | | | |
|---|---|---|---|
| HER-2 Signaling in Breast Cancer | 3.9 | 0.000126 | 0.802 |
| Synaptic Long Term Depression | 3.81 | 0.000155 | 0.866 |
| Acute Phase Response Signaling | 3.8 | 0.000158 | -0.457 |
| EGF Signaling | 3.8 | 0.000158 | 1.091 |
| Wound Healing Signaling Pathway | 3.8 | 0.000158 | 1.408 |
| Melanocyte Development and Pigmentation Signaling | 3.79 | 0.000162 | 0.756 |
| Xenobiotic Metabolism General Signaling Pathway | 3.77 | 0.00017 | 1.093 |
| Autophagy | 3.74 | 0.000182 | -1.336 |
| ERK5 Signaling | 3.74 | 0.000182 | 0.408 |
| Paxillin Signaling | 3.73 | 0.000186 | 0.557 |
| IL-17A Signaling in Airway Cells | 3.64 | 0.000229 | 0.853 |
| UVB-Induced MAPK Signaling | 3.59 | 0.000257 | 1.606 |
| Signaling by Rho Family GTPases | 3.56 | 0.000275 | 0.566 |
| cAMP-mediated signaling | 3.56 | 0.000275 | 0.674 |
| Role of MAPK Signaling in Inhibiting the Pathogenesis of Influenza | 3.53 | 0.000295 | 1.043 |
| G$\alpha$12/13 Signaling | 3.53 | 0.000295 | 1.151 |
| NF-κB Activation by Viruses | 3.53 | 0.000295 | 1.4 |
| Glioma Invasiveness Signaling | 3.53 | 0.000295 | 1.964 |
| Amyotrophic Lateral Sclerosis Signaling | 3.5 | 0.000316 | 0.2 |
| Cardiac β-adrenergic Signaling | 3.48 | 0.000331 | 1.46 |
| Polyamine Regulation in Colon Cancer | 3.43 | 0.000372 | 0.655 |
| Estrogen Receptor Signaling | 3.42 | 0.00038 | 0.113 |
| Induction of Apoptosis by HIV1 | 3.42 | 0.00038 | 1.279 |
| Sumoylation Pathway | 3.41 | 0.000389 | 0.6 |
| 3-phosphoinositide Biosynthesis | 3.39 | 0.000407 | 1.809 |
| $\alpha$-Adrenergic Signaling | 3.37 | 0.000427 | 1.807 |
| ILK Signaling | 3.34 | 0.000457 | 0.152 |

| | | | |
|---|---|---|---|
| Neuregulin Signaling | 3.34 | 0.000457 | 0.557 |
| Corticotropin Releasing Hormone Signaling | 3.34 | 0.000457 | 0.822 |
| CNTF Signaling | 3.32 | 0.000479 | 0.688 |
| Ephrin B Signaling | 3.32 | 0.000479 | 1.213 |
| RHOGDI Signaling | 3.3 | 0.000501 | -1.265 |
| Apelin Muscle Signaling Pathway | 3.23 | 0.000589 | -1.134 |
| Regulation of IL-2 Expression in Activated and Anergic T Lymphocytes | 3.21 | 0.000617 | -0.186 |
| Role Of Osteoblasts In Rheumatoid Arthritis Signaling Pathway | 3.2 | 0.000631 | -2.889 |
| ERBB4 Signaling | 3.2 | 0.000631 | 0.447 |
| RANK Signaling in Osteoclasts | 3.19 | 0.000646 | 0.756 |
| Superpathway of Inositol Phosphate Compounds | 3.15 | 0.000708 | 1 |
| Amyloid Processing | 3.1 | 0.000794 | -0.333 |
| Insulin Receptor Signaling | 3.1 | 0.000794 | 0.822 |
| Adrenomedullin signaling pathway | 3.05 | 0.000891 | -0.146 |
| CCR5 Signaling in Macrophages | 3.02 | 0.000955 | 1.732 |
| D-myo-inositol (1,4,5,6)-Tetrakisphosphate Biosynthesis | 3.02 | 0.000955 | 1.808 |
| D-myo-inositol (3,4,5,6)-tetrakisphosphate Biosynthesis | 3.02 | 0.000955 | 1.808 |
| UVC-Induced MAPK Signaling | 2.98 | 0.001047 | 2.183 |
| VEGF Signaling | 2.97 | 0.001072 | 0.784 |
| Actin Cytoskeleton Signaling | 2.95 | 0.001122 | 1.043 |
| HOTAIR Regulatory Pathway | 2.95 | 0.001122 | 1.406 |
| Role of NFAT in Regulation of the Immune Response | 2.94 | 0.001148 | -0.632 |
| Sperm Motility | 2.93 | 0.001175 | 0.784 |
| Gαs Signaling | 2.93 | 0.001175 | 0.816 |
| D-myo-inositol-5-phosphate Metabolism | 2.91 | 0.00123 | 1.265 |
| Role of p14/p19ARF in Tumor Suppression | 2.9 | 0.001259 | -0.302 |
| IL-15 Signaling | 2.87 | 0.001349 | 0.6 |

| | | | |
|---|---|---|---|
| PI3K Signaling in B Lymphocytes | 2.84 | 0.001445 | -1.029 |
| Prolactin Signaling | 2.83 | 0.001479 | 0.853 |
| Erythropoietin Signaling Pathway | 2.83 | 0.001479 | 1.809 |
| RAC Signaling | 2.81 | 0.001549 | 1.219 |
| Glutamate Receptor Signaling | 2.79 | 0.001622 | -0.447 |
| Pyridoxal 5'-phosphate Salvage Pathway | 2.79 | 0.001622 | -0.229 |
| CD27 Signaling in Lymphocytes | 2.76 | 0.001738 | -0.229 |
| Death Receptor Signaling | 2.75 | 0.001778 | 0.962 |
| Coronavirus Pathogenesis Pathway | 2.73 | 0.001862 | 1.043 |
| Leptin Signaling in Obesity | 2.65 | 0.002239 | 0.577 |
| GPCR-Mediated Nutrient Sensing in Enteroendocrine Cells | 2.63 | 0.002344 | -0.365 |
| DNA Methylation and Transcriptional Repression Signaling | 2.63 | 0.002344 | 0.626 |
| GPCR-Mediated Integration of Enteroendocrine Signaling Exemplified by an L Cell | 2.62 | 0.002399 | 1.606 |
| Th2 Pathway | 2.6 | 0.002512 | 1.414 |
| Thrombopoietin Signaling | 2.58 | 0.00263 | 1.147 |
| Apelin Adipocyte Signaling Pathway | 2.57 | 0.002692 | -1.043 |
| MSP-RON Signaling In Cancer Cells Pathway | 2.53 | 0.002951 | 0.333 |
| Melatonin Signaling | 2.51 | 0.00309 | -0.218 |
| NF-κB Signaling | 2.51 | 0.00309 | 0.762 |
| Pathogen Induced Cytokine Storm Signaling Pathway | 2.48 | 0.003311 | 1.434 |
| ICOS-ICOSL Signaling in T Helper Cells | 2.46 | 0.003467 | -0.962 |
| RHOA Signaling | 2.44 | 0.003631 | 1.257 |
| PD-1, PD-L1 cancer immunotherapy pathway | 2.42 | 0.003802 | -0.853 |
| Phospholipase C Signaling | 2.41 | 0.00389 | 1.18 |
| VDR/RXR Activation | 2.4 | 0.003981 | 0.5 |
| Nitric Oxide Signaling in the Cardiovascular System | 2.4 | 0.003981 | 1.043 |
| CTLA4 Signaling in Cytotoxic T Lymphocytes | 2.39 | 0.004074 | -0.59 |

| | | | |
|---|---|---|---|
| Type I Diabetes Mellitus Signaling | 2.39 | 0.004074 | 0.408 |
| 3-phosphoinositide Degradation | 2.39 | 0.004074 | 1.808 |
| FcγRIIB Signaling in B Lymphocytes | 2.36 | 0.004365 | 0.775 |
| Estrogen-Dependent Breast Cancer Signaling | 2.33 | 0.004677 | 0.894 |
| Role of Hypercytokinemia/hyperchemokinemia in the Pathogenesis of Influenza | 2.32 | 0.004786 | -0.894 |
| PPARα/RXRα Activation | 2.25 | 0.005623 | -0.649 |
| Aryl Hydrocarbon Receptor Signaling | 2.22 | 0.006026 | 0.186 |
| Regulation of Actin-based Motility by Rho | 2.2 | 0.00631 | 1.4 |
| Neuropathic Pain Signaling In Dorsal Horn Neurons | 2.19 | 0.006457 | -0.577 |
| SPINK1 General Cancer Pathway | 2.16 | 0.006918 | 0.5 |
| Role Of Chondrocytes In Rheumatoid Arthritis Signaling Pathway | 2.15 | 0.007079 | -0.507 |
| Neurovascular Coupling Signaling Pathway | 2.14 | 0.007244 | 0.566 |
| Cell Cycle: G2/M DNA Damage Checkpoint Regulation | 2.13 | 0.007413 | 1.604 |
| Role of WNT/GSK-3β Signaling in the Pathogenesis of Influenza | 2.13 | 0.007413 | 1.897 |
| PFKFB4 Signaling Pathway | 2.12 | 0.007586 | -1.604 |
| Relaxin Signaling | 2.12 | 0.007586 | 1.279 |
| Basal Cell Carcinoma Signaling | 2.08 | 0.008318 | 0.832 |
| Osteoarthritis Pathway | 2.02 | 0.00955 | 1.043 |
| Regulation of eIF4 and p70S6K Signaling | 2.01 | 0.009772 | 0.655 |
| Tumoricidal Function of Hepatic Natural Killer Cells | 1.99 | 0.010233 | 0.378 |
| MSP-RON Signaling In Macrophages Pathway | 1.99 | 0.010233 | 0.378 |
| Role of Pattern Recognition Receptors in Recognition of Bacteria and Viruses | 1.95 | 0.01122 | -0.426 |
| Netrin Signaling | 1.93 | 0.011749 | -1.342 |
| CD28 Signaling in T Helper Cells | 1.91 | 0.012303 | 0.756 |
| Toll-like Receptor Signaling | 1.9 | 0.012589 | -1.291 |
| Antiproliferative Role of Somatostatin Receptor 2 | 1.9 | 0.012589 | 1.291 |
| PPAR Signaling | 1.89 | 0.012882 | 0.784 |

| | | | |
|---|---|---|---|
| IL-22 Signaling | 1.86 | 0.013804 | 0.333 |
| nNOS Signaling in Neurons | 1.82 | 0.015136 | -0.378 |
| ATM Signaling | 1.81 | 0.015488 | 1.279 |
| Oncostatin M Signaling | 1.79 | 0.016218 | -0.577 |
| 4-1BB Signaling in T Lymphocytes | 1.75 | 0.017783 | 0.905 |
| Apelin Liver Signaling Pathway | 1.74 | 0.018197 | 1.667 |
| Role of CHK Proteins in Cell Cycle Checkpoint Control | 1.72 | 0.019055 | 0.277 |
| Dendritic Cell Maturation | 1.71 | 0.019498 | -0.973 |
| T Cell Receptor Signaling | 1.71 | 0.019498 | 0.149 |
| Fcγ Receptor-mediated Phagocytosis in Macrophages and Monocytes | 1.71 | 0.019498 | 1.225 |
| Antioxidant Action of Vitamin C | 1.69 | 0.020417 | -0.218 |
| Type II Diabetes Mellitus Signaling | 1.68 | 0.020893 | 0.408 |
| Androgen Signaling | 1.67 | 0.02138 | 0.688 |
| GP6 Signaling Pathway | 1.66 | 0.021878 | 3.157 |
| Antiproliferative Role of TOB in T Cell Signaling | 1.65 | 0.022387 | -0.905 |
| SNARE Signaling Pathway | 1.65 | 0.022387 | 0.18 |
| White Adipose Tissue Browning Pathway | 1.56 | 0.027542 | 0.707 |
| Lymphotoxin β Receptor Signaling | 1.54 | 0.02884 | 0.535 |
| Pyroptosis Signaling Pathway | 1.53 | 0.029512 | -0.655 |
| Growth Hormone Signaling | 1.53 | 0.029512 | 1 |
| Apelin Cardiac Fibroblast Signaling Pathway | 1.5 | 0.031623 | -1.414 |
| MIF Regulation of Innate Immunity | 1.5 | 0.031623 | 0.577 |
| NRF2-mediated Oxidative Stress Response | 1.49 | 0.032359 | 0.898 |
| Regulation of Cellular Mechanics by Calpain Protease | 1.47 | 0.033884 | -0.535 |
| Apelin Pancreas Signaling Pathway | 1.46 | 0.034674 | -0.832 |
| Dermatan Sulfate Biosynthesis | 1.43 | 0.037154 | 0.535 |
| eNOS Signaling | 1.43 | 0.037154 | 1.3 |

| | | | |
|---|---|---|---|
| Dermatan Sulfate Biosynthesis (Late Stages) | 1.39 | 0.040738 | 0.302 |
| Heparan Sulfate Biosynthesis | 1.39 | 0.040738 | 0.688 |
| IL-17A Signaling in Gastric Cells | 1.39 | 0.040738 | 0.816 |
| Inhibition of ARE-Mediated mRNA Degradation Pathway | 1.36 | 0.043652 | 0.354 |
| CD40 Signaling | 1.31 | 0.048978 | 1 |
| Activation of IRF by Cytosolic Pattern Recognition Receptors | 1.3 | 0.050119 | -0.535 |
