# Supplementary material for: Circulating Total Extracellular Vesicles Cargo Are Associated with Age-Related Oxidative Stress and Susceptibility to Cardiovascular Diseases: Exploratory Results from Microarray Data
Source: Biomedicines. 2023 Oct 28;11(11):2920. doi: 10.3390/biomedicines11112920 (PMC10669226; doi:10.3390/biomedicines11112920)
Supplement: Supplementary file 1 [file biomedicines-11-02920-s001.zip › Supplementary Table S3.pdf]

Supplementary Table S3. Cardiac Hypertrophy Canonical Pathway

miRNA	Symbol	Gene Name	p-value	Molecule Predicted Fold Change	Location
miR-92a-3p	ADCY3	adenylate cyclase 3	0.00168	1.416	Plasma Membrane
miR-92a-3p	ADRB1	adrenoceptor beta 1	0.00168	1.416	Plasma Membrane
miR-92a-3p	GNAQ	G protein subunit alpha q	0.00168	1.416	Plasma Membrane
miR-92a-3p	HAND1	heart and neural crest derivatives expressed 1	0.00168	1.416	Nucleus
miR-92a-3p	HAND2	heart and neural crest derivatives expressed 2	0.00168	1.416	Nucleus
miR-92a-3p	MAP2K4	mitogen-activated protein kinase kinase 4	0.00168	1.416	Cytoplasm
miR-92a-3p	PIK3R3	phosphoinositide-3-kinase regulatory subunit 3	0.00168	1.416	Cytoplasm
miR-92a-3p	RAP1B	RAP1B, member of RAS oncogene family	0.00168	1.416	Cytoplasm
miR-31-3p	PIK3CG	phosphatidylinositol-4,5-bisphosphate 3-kinase catalytic subunit gamma	0.0436	1.339	Cytoplasm
miR-190a-5p	CACNB2	calcium voltage-gated channel auxiliary subunit beta 2	0.0107	1.335	Plasma Membrane
miR-190a-5p	GUCY1B1	guanylate cyclase 1 soluble subunit beta 1	0.0107	1.335	Cytoplasm
miR-30c-1-3p	CACNG7	calcium voltage-gated channel auxiliary subunit gamma 7	0.0424	1.298	Plasma Membrane
miR-30c-1-3p	CHP1	calcineurin like EF-hand protein 1	0.0424	1.298	Cytoplasm
miR-30c-1-3p	MAPK13	mitogen-activated protein kinase 13	0.0424	1.298	Cytoplasm
miR-30c-1-3p	PIK3R6	phosphoinositide-3-kinase regulatory subunit 6	0.0424	1.298	Cytoplasm
miR-30c-1-3p	RALB	RAS like proto-oncogene B	0.0424	1.298	Cytoplasm
miR-30c-1-3p	RHOJ	ras homolog family member J	0.0424	1.298	Cytoplasm
miR-30c-1-3p	ROCK1	Rho associated coiled-coil containing protein kinase 1	0.0424	1.298	Cytoplasm
miR-30c-1-3p	EIF4E	eukaryotic translation initiation factor 4E	0.0486	1.286	Cytoplasm
miR-30c-1-3p	GNB1	G protein subunit beta 1	0.0486	1.286	Plasma Membrane
miR-30c-1-3p	MAPK8	mitogen-activated protein kinase 8	0.0486	1.286	Cytoplasm
miR-30c-1-3p	NRAS	NRAS proto-oncogene, GTPase	0.0486	1.286	Plasma Membrane
miR-34a-5p	ADRA1D	adrenoceptor alpha 1D	0.00859	1.276	Plasma Membrane
miR-34a-5p	CACNB3	calcium voltage-gated channel auxiliary subunit beta 3	0.00859	1.276	Plasma Membrane
miR-34a-5p	CACNG8	calcium voltage-gated channel auxiliary subunit gamma 8	0.00859	1.276	Plasma Membrane
miR-34a-5p	GNAO1	G protein subunit alpha o1	0.00859	1.276	Plasma Membrane
miR-34a-5p	GNAS	GNAS complex locus	0.00859	1.276	Plasma Membrane
miR-34a-5p	MAP2K1	mitogen-activated protein kinase kinase 1	0.00859	1.276	Cytoplasm
miR-34a-5p	RRAS	RAS related	0.00859	1.276	Cytoplasm
miR-22-3p	GNAI3	G protein subunit alpha i3	0.0351	1.272	Cytoplasm
miR-22-3p	MAPK14	mitogen-activated protein kinase 14	0.0351	1.272	Cytoplasm
miR-22-3p	RHOV	ras homolog family member V	0.0351	1.272	Plasma Membrane
miR-22-3p	SRF	serum response factor	0.0351	1.272	Nucleus
miR-3585-5p	RHOA	ras homolog family member A	0.00394	1.251	Cytoplasm
miR-181a-1-3p	MAP3K5	mitogen-activated protein kinase kinase kinase 5	0.00689	1.241	Cytoplasm

miR-199a-3p	ADCY9	adenylate cyclase 9	0.00331	1.233	Plasma Membrane
miR-199a-3p	GNB2	G protein subunit beta 2	0.00331	1.233	Plasma Membrane
miR-199a-3p	MAP3K11	mitogen-activated protein kinase kinase kinase 11	0.00331	1.233	Cytoplasm
miR-199a-3p	RAC1	Rac family small GTPase 1	0.00331	1.233	Plasma Membrane
miR-199a-3p	ROCK2	Rho associated coiled-coil containing protein kinase 2	0.00331	1.233	Cytoplasm
miR-199a-3p	TGFB2	transforming growth factor beta 2	0.00331	1.233	Extracellular Space
miR-199a-3p	TGFBR1	transforming growth factor beta receptor 1	0.00331	1.233	Plasma Membrane
let-7a-5p	ADRB2	adrenoceptor beta 2	0.0378	1.16	Plasma Membrane
let-7a-5p	ADRB3	adrenoceptor beta 3	0.0378	1.16	Plasma Membrane
let-7a-5p	GNG5	G protein subunit gamma 5	0.0378	1.16	Plasma Membrane
let-7a-5p	IGF1R	insulin like growth factor 1 receptor	0.0378	1.16	Plasma Membrane
let-7a-5p	MAP3K13	mitogen-activated protein kinase kinase kinase 13	0.0378	1.16	Cytoplasm
let-7a-5p	PRKAR2A	protein kinase cAMP-dependent type II regulatory subunit alpha	0.0378	1.16	Cytoplasm
let-7a-5p	RHOB	ras homolog family member B	0.0378	1.16	Cytoplasm
let-7a-5p	RHOG	ras homolog family member G	0.0378	1.16	Cytoplasm
miR-410-5p	CACNA2D 1	calcium voltage-gated channel auxiliary subunit alpha2delta 1	0.033	-1.154	Plasma Membrane
miR-410-5p	RHOBTB2	Rho related BTB domain containing 2	0.033	-1.154	Cytoplasm
miR-143-3p	KRAS	KRAS proto-oncogene, GTPase	0.0194	-1.212	Cytoplasm
miR-143-3p	MAPK12	mitogen-activated protein kinase 12	0.0194	-1.212	Cytoplasm
miR-142-3p	GNA12	G protein subunit alpha 12	0.0423	-1.233	Plasma Membrane
miR-142-3p	MAP3K4	mitogen-activated protein kinase kinase kinase 4	0.0423	-1.233	Cytoplasm
miR-142-3p	MTOR	mechanistic target of rapamycin kinase	0.0423	-1.233	Nucleus
miR-142-3p	PIK3CB	phosphatidylinositol-4,5-bisphosphate 3-kinase catalytic subunit beta	0.0423	-1.233	Cytoplasm
miR-17-5p	GUCY1A1	guanylate cyclase 1 soluble subunit alpha 1	0.0462	-1.249	Cytoplasm
miR-17-5p	MAP3K2	mitogen-activated protein kinase kinase kinase 2	0.0462	-1.249	Cytoplasm
miR-17-5p	MAP3K12	mitogen-activated protein kinase kinase kinase 12	0.0462	-1.249	Cytoplasm
miR-17-5p	MEF2D	myocyte enhancer factor 2D	0.0462	-1.249	Nucleus
miR-17-5p	PPP3R1	protein phosphatase 3 regulatory subunit B, alpha	0.0462	-1.249	Cytoplasm
miR-17-5p	RHOC	ras homolog family member C	0.0462	-1.249	Plasma Membrane
miR-214-3p	ADSS2	adenylosuccinate synthase 2	0.0334	-1.265	Cytoplasm
miR-214-3p	ATF2	activating transcription factor 2	0.0334	-1.265	Nucleus
miR-214-3p	MAP3K9	mitogen-activated protein kinase kinase kinase 9	0.0334	-1.265	Cytoplasm
miR-296-3p	CREB1	cAMP responsive element binding protein 1	0.0226	-1.288	Nucleus
miR-296-3p	PPP3R2	protein phosphatase 3 regulatory subunit B, beta	0.0226	-1.288	Cytoplasm
miR-296-3p	PRKAG1	protein kinase AMP-activated non-catalytic subunit gamma 1	0.0226	-1.288	Nucleus
miR-153-3p	GRB2	growth factor receptor bound protein 2	0.0226	-1.382	Cytoplasm
miR-153-3p	PLCB1	phospholipase C beta 1	0.0226	-1.382	Cytoplasm
miR-153-3p	PLCZ1	phospholipase C zeta 1	0.0226	-1.382	Cytoplasm

miR-153-3p	TGFBR2	transforming growth factor beta receptor 2	0.0226	-1.382	Plasma Membrane
miR-150-3p	GNG12	G protein subunit gamma 12	0.0297	-1.401	Plasma Membrane
miR-150-3p	RAP2B	RAP2B, member of RAS oncogene family	0.0297	-1.401	Plasma Membrane
miR-128-1-5p	CACNA1A	calcium voltage-gated channel subunit alpha1 A	0.0468	-1.906	Plasma Membrane
miR-128-1-5p	CACNG4	calcium voltage-gated channel auxiliary subunit gamma 4	0.0468	-1.906	Plasma Membrane
miR-128-1-5p	GNAI2	G protein subunit alpha i2	0.0468	-1.906	Plasma Membrane
miR-128-1-5p	GNAZ	G protein subunit alpha z	0.0468	-1.906	Plasma Membrane
miR-128-1-5p	GNG13	G protein subunit gamma 13	0.0468	-1.906	Plasma Membrane
miR-128-1-5p	MAP2K3	mitogen-activated protein kinase kinase 3	0.0468	-1.906	Cytoplasm
miR-128-1-5p	MEF2B	myocyte enhancer factor 2B	0.0468	-1.906	Nucleus
miR-128-1-5p	NKX2-5	NK2 homeobox 5	0.0468	-1.906	Nucleus
miR-128-1-5p	NOTUM	notum, palmitoleoyl-protein carboxylesterase	0.0468	-1.906	Extracellular Space
miR-128-1-5p	PIK3R2	phosphoinositide-3-kinase regulatory subunit 2	0.0468	-1.906	Cytoplasm
miR-128-1-5p	PLCH2	phospholipase C eta 2	0.0468	-1.906	Cytoplasm
miR-128-1-5p	PRKACA	protein kinase cAMP-activated catalytic subunit alpha	0.0468	-1.906	Cytoplasm
miR-128-1-5p	RASD2	RASD family member 2	0.0468	-1.906	Cytoplasm
miR-128-1-5p	TGFB1	transforming growth factor beta 1	0.0468	-1.906	Extracellular Space
