# Supplementary material for: Circulating Total Extracellular Vesicles Cargo Are Associated with Age-Related Oxidative Stress and Susceptibility to Cardiovascular Diseases: Exploratory Results from Microarray Data
Source: Biomedicines. 2023 Oct 28;11(11):2920. doi: 10.3390/biomedicines11112920 (PMC10669226; doi:10.3390/biomedicines11112920)
Supplement: Supplementary file 1 [file biomedicines-11-02920-s001.zip › Supplementary Table S4.pdf]

Supplementary Table S4.

Ingenuity Canonical Pathways	p-value	-log(p-value)	Ratio	z-score	miRNAs targetting pathway	Molecules
Production of Nitric Oxide and Reactive Oxygen Species in Macrophages	1.2E-06	5.92	0.318	0.412	19	56
Nitric Oxide Signaling in the Cardiovascular System	0.004	2.4	0.274	1.043	15	31
nNOS Signaling in Neurons	0.0151	1.82	0.311	-0.378	9	14
Antioxidant Action of Vitamin C	0.0204	1.69	0.255	-0.218	13	26
NRF2-mediated Oxidative Stress Response	0.0323	1.49	0.221	0.898	19	49
eNOS Signaling	0.0371	1.43	0.231	1.3	16	34

Canonical pathways are ranked according to –log(p-value), calculated by Fisher's exact test right-tailed.
