# Supplementary material for: Circulating Total Extracellular Vesicles Cargo Are Associated with Age-Related Oxidative Stress and Susceptibility to Cardiovascular Diseases: Exploratory Results from Microarray Data
Source: Biomedicines. 2023 Oct 28;11(11):2920. doi: 10.3390/biomedicines11112920 (PMC10669226; doi:10.3390/biomedicines11112920)
Supplement: Supplementary file 1 [file biomedicines-11-02920-s001.zip › Supplementary Table S5.pdf]

Supplementary Table S5. Production of Nitric Oxide and Reactive Oxygen Species Macrophages Canonical Pathway

miRNA	Symbol	Gene Name	p-value	Molecule Predicted Fold Change	Location
miR-128-1-5p	AKT2	AKT serinethreonine kinase 2	0.0468	-1.906	Cytoplasm
miR-22-3p	AKT3	AKT serinethreonine kinase 3	0.0351	1.272	Cytoplasm
miR-128-1-5p	APOC2	apolipoprotein C2	0.0468	-1.906	Extracellular Space
miR-3064-5p	APOC4	apolipoprotein C4	0.0478	-1.148	Extracellular Space
let-7a-5p	ARG2	arginase 2	0.0378	1.16	Cytoplasm
miR-190a-5p	CAT	catalase	0.0107	1.335	Cytoplasm
miR-128-1-5p	CLU	clusterin	0.0468	-1.906	Cytoplasm
miR-128-1-5p	IKBKB	inhibitor of nuclear factor kappa B kinase subunit beta	0.0468	-1.906	Cytoplasm
miR-34a-5p	IKBKE	inhibitor of nuclear factor kappa B kinase subunit epsilon	0.00859	1.276	Cytoplasm
miR-128-1-5p	IKBKG	inhibitor of nuclear factor kappa B kinase regulatory subunit gamma	0.0468	-1.906	Nucleus
miR-17-5p	JAK1	Janus kinase 1	0.0462	-1.249	Cytoplasm
miR-34a-5p	MAP2K1	mitogen-activated protein kinase kinase 1	0.00859	1.276	Cytoplasm
miR-92a-3p	MAP2K4	mitogen-activated protein kinase kinase 4	0.00168	1.416	Cytoplasm
miR-142-3p	MAP3K11	mitogen-activated protein kinase kinase kinase 11	0.00331	1.233	Cytoplasm
miR-17-5p	MAP3K12	mitogen-activated protein kinase kinase kinase 12	0.0462	-1.249	Cytoplasm
let-7a-5p	MAP3K13	mitogen-activated protein kinase kinase kinase 13	0.0378	1.16	Cytoplasm
miR-17-5p	MAP3K2	mitogen-activated protein kinase kinase kinase 2	0.0462	-1.249	Cytoplasm
miR-199a-3p	MAP3K4	mitogen-activated protein kinase kinase kinase 4	0.0423	-1.233	Cytoplasm
miR-181a-1-3p	MAP3K5	mitogen-activated protein kinase kinase kinase 5	0.00689	1.241	Cytoplasm
miR-214-3p	MAP3K9	mitogen-activated protein kinase kinase kinase 9	0.0334	-1.265	Cytoplasm
miR-143-3p	MAPK12	mitogen-activated protein kinase 12	0.0194	-1.212	Cytoplasm
miR-30c-1-3p	MAPK13	mitogen-activated protein kinase 13	0.0424	1.298	Cytoplasm
miR-22-3p	MAPK14	mitogen-activated protein kinase 14	0.0351	1.272	Cytoplasm

miR-3120	MAPK8	mitogen-activated protein kinase 8	0.0486	1.286	Cytoplasm
miR-128-1-5p	NGFR	nerve growth factor receptor	0.0468	-1.906	Plasma Membrane
miR-199a-3p	PIK3CB	phosphatidylinositol-4,5-bisphosphate 3-kinase catalytic subunit beta	0.0423	-1.233	Cytoplasm
miR-31-3p	PIK3CG	phosphatidylinositol-4,5-bisphosphate 3-kinase catalytic subunit gamma	0.0436	1.339	Cytoplasm
miR-128-1-5p	PIK3R2	phosphoinositide-3-kinase regulatory subunit 2	0.0468	-1.906	Cytoplasm
miR-92a-3p	PIK3R3	phosphoinositide-3-kinase regulatory subunit 3	0.00168	1.416	Cytoplasm
miR-30c-1-3p	PIK3R6	phosphoinositide-3-kinase regulatory subunit 6	0.0424	1.298	Cytoplasm
miR-22-3p	PPARA	peroxisome proliferator activated receptor alpha	0.0351	1.272	Nucleus
miR-34a-5p	PPP1R14D	protein phosphatase 1 regulatory inhibitor subunit 14D	0.00859	1.276	Cytoplasm
miR-214-3p	PPP1R3D	protein phosphatase 1 regulatory subunit 3D	0.0334	-1.265	Cytoplasm
let-7a-5p	PPP1R7	protein phosphatase 1 regulatory subunit 7	0.0378	1.16	Nucleus
miR-3585-5p	PPP2CA	protein phosphatase 2 catalytic subunit alpha	0.00394	1.251	Cytoplasm
miR-142-3p	PPP2R1A	protein phosphatase 2 scaffold subunit Aalpha	0.00331	1.233	Cytoplasm
miR-199a-3p	PPP2R2A	protein phosphatase 2 regulatory subunit Balpha	0.0423	-1.233	Cytoplasm
miR-150-3p	PPP2R3A	protein phosphatase 2 regulatory subunit B''alpha	0.0297	-1.401	Nucleus
miR-199a-3p	PPP2R5E	protein phosphatase 2 regulatory subunit B'epsilon	0.0423	-1.233	Cytoplasm
miR-142-3p	PRKCA	protein kinase C alpha	0.00331	1.233	Cytoplasm
miR-22-3p	PRKCB	protein kinase C beta	0.0351	1.272	Cytoplasm
miR-30c-1-3p	PRKCG	protein kinase C gamma	0.0424	1.298	Cytoplasm
miR-128-1-5p	PRKCQ	protein kinase C theta	0.0468	-1.906	Cytoplasm
miR-142-3p	RAC1	Rac family small GTPase 1	0.00331	1.233	Plasma Membrane
miR-92a-3p	RAP1B	RAP1B, member of RAS oncogene family	0.00168	1.416	Cytoplasm
miR-3585-5p	RHOA	ras homolog family member A	0.00394	1.251	Cytoplasm
let-7a-5p	RHOB	ras homolog family member B	0.0378	1.16	Cytoplasm
miR-410-5p	RHOBTB2	Rho related BTB domain containing 2	0.033	-1.154	Cytoplasm
miR-17-5p	RHOC	ras homolog family member C	0.0462	-1.249	Plasma Membrane
let-7a-5p	RHOG	ras homolog family member G	0.0378	1.16	Cytoplasm

miR-30c-1-3p	RHOJ	ras homolog family member J	0.0424	1.298	Cytoplasm
miR-22-3p	RHOV	ras homolog family member V	0.0351	1.272	Plasma Membrane
let-7a-5p	S100A8	S100 calcium binding protein A8	0.0378	1.16	Cytoplasm
miR-128-1-5p	SIRPA	signal regulatory protein alpha	0.0468	-1.906	Plasma Membrane
let-7a-5p	TLR4	toll like receptor 4	0.0378	1.16	Plasma Membrane
miR-17-5p	TNF	tumor necrosis factor	0.0462	-1.249	Extracellular Space
